# Resection of a methicillin-resistant *Staphylococcus aureus* liver abscess in a patient with Crohn’s disease under infliximab treatment: a case report

**DOI:** 10.1186/1752-1947-7-36

**Published:** 2013-02-01

**Authors:** Junichi Togashi, Yasuhiko Sugawara, Nobuhisa Akamatsu, Taku Aoki, Masayoshi Ijichi, Mami Tanabe, Koji Kusaka, Masayuki Shibazaki, Tokuma Tadami, Minako Sakou, Masakazu Takazoe, Yasutsugu Bandai, Norihiro Kokudo

**Affiliations:** 1 Department of Surgery, Artificial Organ and Transplantation Surgery Division, University of Tokyo, 7-3-1 Hongo, Bunkyo-ku, Tokyo, 113-8655, Japan; 2 Department of Surgery, Central Hospital of Social Health Insurance, 3-22-1 Hyakunin-cho, Shinjuku-ku, Tokyo, 169-0073, Japan; 3 Department of Hepato-Biliary-Pancreatic Surgery, Saitama Medical Center, Saitama Medical University, 1981, Kamoda, Kawagoe, Saitama, 350-8550, Japan; 4 Department of Internal Medicine, Central Hospital of Social Health Insurance, 3-22-1 Hyakunin-cho, Shinjuku-ku, Tokyo, 169-0073, Japan

## Abstract

**Introduction:**

A liver abscess in Crohn’s disease is a rare but important entity that is associated with a poor prognosis and high mortality when treatment is delayed. We report a case of successful liver segmentectomy for a methicillin-resistant *Staphylococcus aureus* liver abscess in a patient with Crohn’s disease under infliximab treatment.

**Case presentation:**

A 31-year-old Japanese man, who had been treated with infliximab infusions for Crohn’s disease, was referred to our hospital presenting with an abrupt onset of high fever and an elevated white blood cell count and serum C-reactive protein level. Computed tomography revealed a liver abscess occupying segment 8. The limited effect of percutaneous transhepatic abscess drainage and antibiotics led us to perform radical resection of the abscess. The patient recovered quickly after surgery and the postoperative course was uneventful.

**Conclusion:**

The present case suggests that surgical removal of an abscess should be considered for patients under immunosuppression or refractory to conventional treatment.

## Introduction

Infliximab treatment has recently emerged as a safe and effective method of inducing and maintaining remission of Crohn’s disease [1-3], but the immunosuppression sometimes results in unexpected complications, such as infection with *Mycobacterium tuberculosis*, sepsis, severe pneumonia, severe liver dysfunction, and leucopenia. Among the potential complications, a liver abscess is critical, with a higher incidence than that in the general population (114 to 297 per 100,000) [4].

Here, we report a case of a large solitary methicillin-resistant *Staphylococcus aureus* (MRSA) liver abscess localized in Couinaud’s segment 8 in a patient with Crohn’s disease under infliximab treatment, which was successfully resected and cured by anatomic liver resection following medical and interventional treatment failure.

## Case presentation

A 31-year-old Japanese man undergoing infliximab treatment for Crohn’s disease was admitted to our hospital with high fever and pain in the right upper abdominal region. He was diagnosed with Crohn’s disease 15 years ago when he had presented with an anal fistula, which was stable without resistant abscess for five years. Due to gradual exacerbation of the disease, he underwent an ileocecal resection with an ileostomy, and subsequent abscess drainage around the stoma 10 years ago. A second operation for ileal stenosis was performed with a partial ileum resection and a new ileostomy six years before the admission. He had not been treated with methylprednisolone, but underwent infliximab treatment two years before the admission to induce remission because of further exacerbation of the disease. Infliximab (five mg/kg) was administered every eight weeks for nine times, successfully suppressing the disease. The last infusion of infliximab was administered two months before referral to our department.

**Figure 1:**
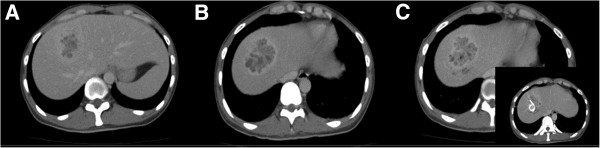
**Computed tomography images of the lesion. A**) Cystic heterogeneous lesion in segment 8 of the right liver lobe, with a partly liquefied component. **B**) Axial images showing a rim-enhancing lesion suggestive of residual liver abscess after drainage and vancomycin treatment. **C**) Axial images showing a rim-enhancing lesion suggestive of residual liver abscess after drainage with a larger tube and teicoplanin treatment.

On admission, his mean arterial pressure, heart rate, body temperature, and respiratory rate were 76mmHg, 78/minute, 39.3°C, and 16/minute, respectively. After admission, his fever spiked up to 40°C each day, however, his circulatory and respiratory condition was stable without vasopressor treatment or oxygen administration. Laboratory data revealed severe inflammation with a white blood cell count of 20400/μL with 80% neutrophils, a serum C-reactive protein level of 25.0mg/dL, a serum fibrin degradation products level of 26μg/mL, and a serum D-dimer level of 11.6μg/mL. His liver function was slightly impaired with increased aspartate and alanine aminotransferase levels (87IU/L and 149IU/L, respectively); however, no other organ impairment was observed. The results of the arterial blood culture and serum anti-ameba shigella dysenteriae antibody tests were negative. Abdominal ultrasonography and abdominal computed tomography on admission revealed a huge heterogeneous lesion in the liver, 50mm in diameter, occupying Couinaud’s segment 8 (Figure 1A).

An eight-French scale (Fr) percutaneous transhepatic abscess drainage (PTAD) tube was inserted and the patient received intravenous sulbactam sodium and cefoperazone sodium (four g/day) and subsequent meropenem (four g/day). The first microbial culture of the pus that drained from the PTAD revealed MRSA and *Escherichia coli*. MRSA was confirmed in MRSA screening agar. The minimum inhibitory concentration of vancomycin was one mg/L. Therefore vancomycin (one g/day) was started and continued for eight days, but the patient’s fever and laboratory and radiographic findings did not improve (Figures 1B and 1C). Vancomycin was then changed to teicoplanin with an initial dose of 400mg/day and a maintenance dose of 200mg/day maintaining the target trough level at 11 to 13μg/mL. Additional interventional treatment with placement of another larger (12Fr) PTAD tube was performed, which failed to relieve the liver abscess, and the size of the abscess increased to 60mm in diameter on the follow-up computed tomography scan (Figure 1C). During the course, additional blood cultures were taken twice and an abscess culture was performed which resulted negative for bacteremia but positive for MRSA abscess. Without any improvement in the patient’s general condition and clinical findings after seven days of teicoplanin treatment with additional interventional treatments, we decided to perform urgent radical resection of the abscess. Under general anesthesia, a liver resection of segment 8 was performed through an inverted T-shaped incision on day 30 after admission.

**Figure 2:**
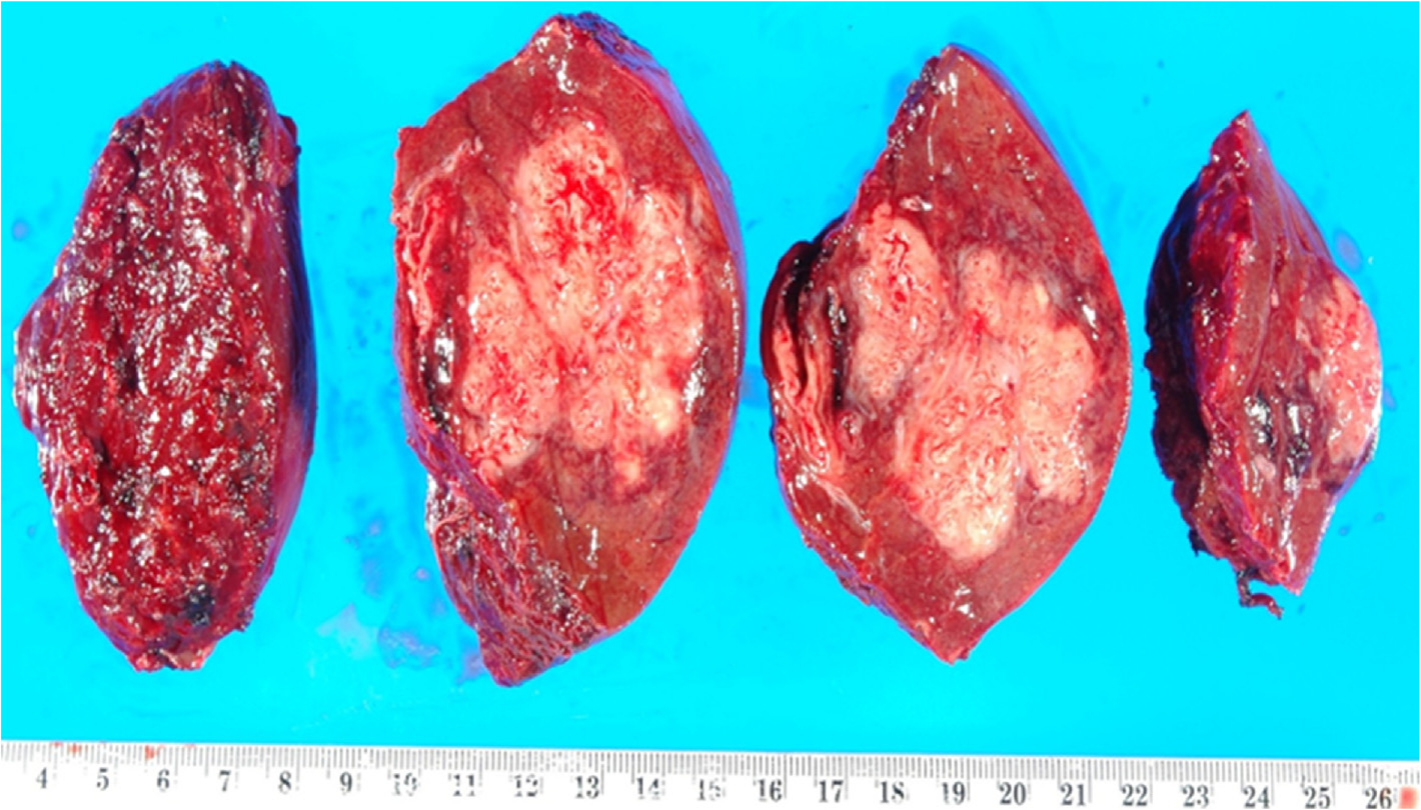
**Resected portion of the liver demonstrating liver abscess, which was positive for methicillin-resistant *Staphylococcus aureus *** **on culture.**

**Figure 3:**
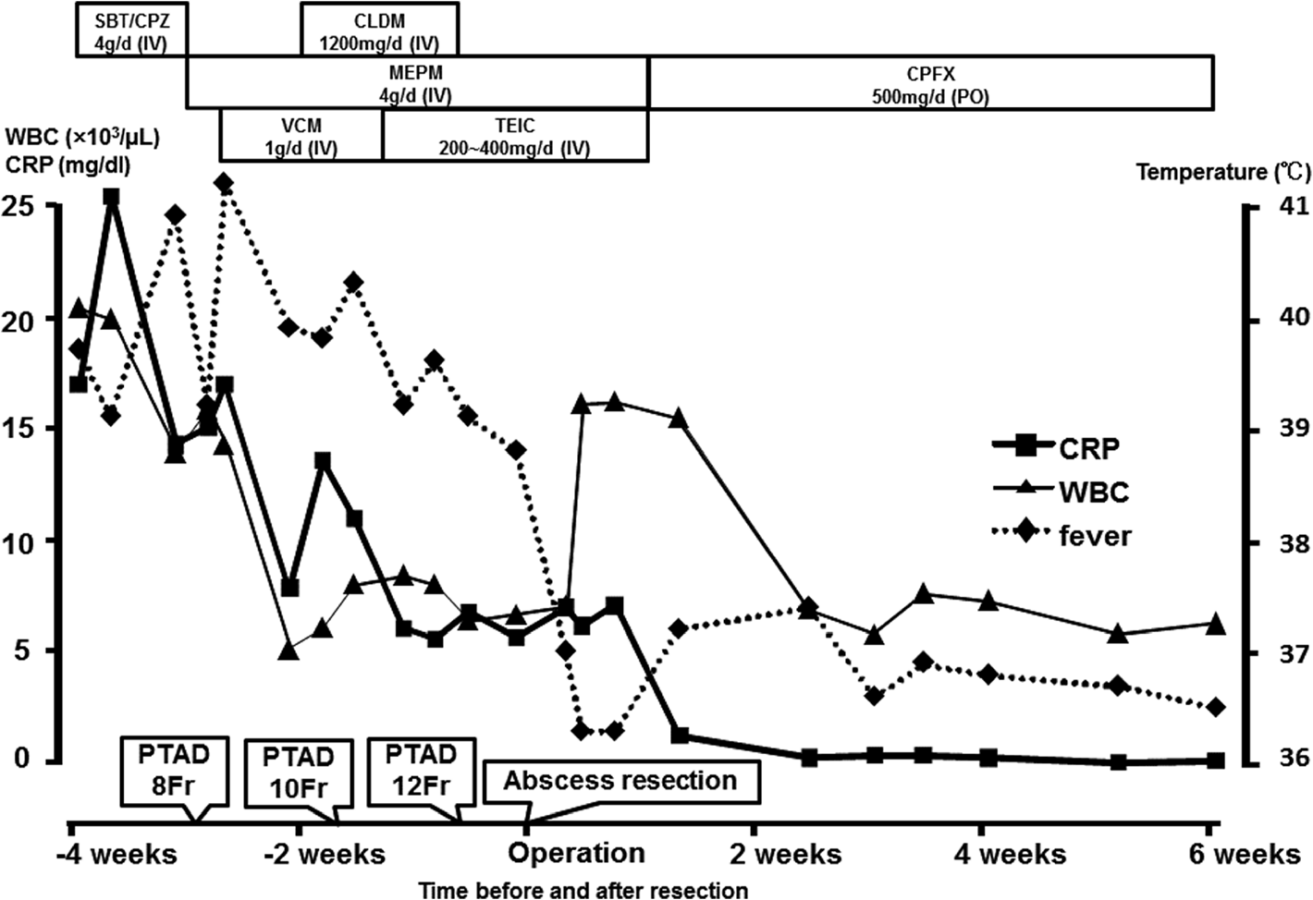
**Clinical course with changes in the laboratory data.** Abbreviations: CLDM, clindamycin; CPFX, ciprofloxacin; MEPM, meropenem; PTAD, percutaneous transhepatic abscess drainage; SBT/CPZ, sulbactam sodium and cefoperazone sodium; TEIC, teicoplanin; WBC, white blood cell counts; CRP, C-reactive protein.

Meticulous guidance by intraoperative ultrasonography allowed us resection of segment 8 without exposing the abscess cavity. The operating time and intra-operative blood loss were 510 minutes and 900mL, respectively. A pathologic examination revealed a liver abscess, 65×45×36mm in size, weighing 200g, composed of irregular necrosis with epithelioid and fibrotic formations in a surrounding palisading pattern (Figure 2). The patient’s temperature returned to normal and the laboratory data also normalized immediately after the operation. The patient received intravenous teicoplanin and meropenem for seven more days after surgery. After that, 500mg/day of ciprofloxacin was administered orally. The postoperative course was uneventful, except for intravenous hyperalimentation catheter-related *Candida parapsilosis*, which was successfully treated with fluconazole. The patient was discharged on postoperative day 41. The treatment course of the patient is summarized in Figure 3 with the changes in the laboratory data. So far, no evidence of recurrent liver abscess or Crohn’s disease progression has been observed in the four months after the resection, with oral administration of three g/day of PENTASA® (mesalamine) and 500mg/day of ciprofloxacin.

## Discussion

Pyogenic liver abscess (PLA) is uncommon with an incidence of 0.5% to 0.8%, accounting for 15 per 100,000 hospital admissions, but it is an important entity due to the potential lethality [5]. Currently, the most common cause of PLA is biliary tract disease, including cholelithiasis, benign and/or malignant biliary strictures, and congenital anomalies of the biliary tree, reported to account for 37% of cases [6,7]. Appendicitis, diverticulitis, inflammatory bowel disease, and perforated hollow viscera are possible sources of septic emboli. Hematogenous dissemination of causative organisms in association with systemic bacteremia, for example from endocarditis or pyelonephritis, is a rare cause of PLA. The bacteriology of a PLA is polymicrobial, with *Klebsiella*, group D *Streptococcus*, *E. coli*, and *Bacteroides*. Although recovery of *S. aureus* from PLA with conservative therapy occurs in approximately 4% of cases, that of MRSA is rare [6].

Inflammatory bowel diseases, including Crohn’s disease, are associated with frequent portal venous bacteremia due to disruption of the bowel barrier, which easily results in bacterial translocation [8]. In addition, the well-known complications of Crohn’s disease, such as fistulization, perforation, and intra-abdominal abscess formation could predispose patients to the development of microbial invasion of the portal venous system and seeding of the hepatic parenchyma [9]. Thus, Crohn’s disease is associated with various hepatobiliary disorders, including pericholangitis, sclerosing cholangitis, granulomatous hepatitis, and cholelithiasis [10], but the development of PLA is rare, with only 47 reported cases in the English literature [8,9,11-13]. Many authors have pointed out that not only bowel inflammation itself but also treatment with steroids or infliximab, malnutrition, and the underlying immunologic impairment of patients with Crohn’s disease might have a role in developing PLA. To the best of our knowledge, this is the first report of a MRSA liver abscess developing in a patient with Crohn’s disease under infliximab treatment. In this particular case, the Crohn’s disease itself was well suppressed with successful remission induced after nine infusions of infliximab. In addition, a colon and intestinal examination before discharge revealed almost normal mucosa without any symptoms. It is also well-accepted that an immunocompromised condition plays a critical role in developing PLA [5,14]. All these findings led us to speculate that the immunosuppressive state under infliximab treatment might have played a critical role in the development of the MRSA liver abscess in the present case, considering the documented association between cytokine inhibition by infliximab and infection [13,15-17].

Systemic parenteral antimicrobial therapy with broad spectrum antibiotics remains the mainstay primary treatment of PLA. The regimen must be altered to target specific organisms isolated from the abscess aspirate [5]. An abscess larger than three cm in diameter is generally required to be drained [17]. The image-guided percutaneous technique is a standard treatment of drainage [7]. Nevertheless, there remains a role for open or laparoscopic surgical intervention in the management of PLA with the documented indication as follows: no clinical response after four to seven days of percutaneous drainage; multiple, large, or loculated abscess; thick-walled abscess with viscous pus; and concomitant intra-abdominal surgical pathology [5,16,18,19]. Some authors have reported the efficacy and safety of emergent surgical drainage or resection [9,10,14,20,21].

Because of its rarity, it is unclear whether PLA caused by *S. aureus* (or MRSA) follows a different or more severe clinical course than PLA caused by enteric flora. The aggressive nature of the present PLA might validate the decision to perform surgery. Considering that few cases of surgical resection of PLA [15] have been reported, the necessity of anatomic resection of PLA including the surrounding parenchyma might be a matter of debate, but the satisfactory course of the present case after resection justifies our decision.

However, there were some critical flaws in our management of this case. First, an expert panel has recently recommended the selection of daptomycin or linezolid as a second- or even first-line treatment for serious MRSA infection [22]. Considering that the crucial points for the treatment of a patient with severe infection are prompt resuscitation management, adequate source control, and proper antibiotic therapy, the way we selected the antibiotic could be criticized as inappropriate in this case. Second, the operative procedure, anatomic segment 8 resection, took too long, which might have burdened some unnecessary stress on the patient. Yet, we would still like to emphasize that the conversion to surgical removal without delay seems crucial in a case with a complicated severe abscess as in the present patient.

## Conclusion

Here we reported a case of a MRSA liver abscess in a patient under infliximab treatment for Crohn’s disease. Resection of a liver abscess could be an option for refractory PLA.

## Consent

Written informed consent was obtained from the patient for publication of this case report and accompanying images. A copy of the written consent is available for review by the Editor-in-Chief of this journal.

## Abbreviations

Fr: French scale; MRSA: Methicillin-resistant *Staphylococcus aureus*; PLA: Pyogenic liver abscess; PTAD: Percutaneous transhepatic abscess drainage.

## Competing interests

The authors declare that they have no competing interests.

## Authors’ contributions

JT, YS, NA, TA, NK, MSh, TT, MSa, MTan, YB and KK collected, analyzed and interpreted the patient data. MI, MTak and KK performed the histological examination of the liver. JT, YS and NA contributed in writing the manuscript. All authors read and approved the final manuscript.

## References

[B1] Hanauer SB Feagan BG Lichtenstein GR Mayer LF Schreiber S Colombel JF Rachmilewitz D Wolf DC Olson A Bao W Rutgeerts P ACCENT I Study Group Maintenance infliximab for Crohn’s disease: the ACCENT I randomised trial Lancet 2002 359 1541 1549 10.1016/S0140-6736(02)08512-4 12047962

[B2] Rutgeerts P Feagan BG Lichtenstein GR Mayer LF Schreiber S Colombel JF Rachmilewitz D Wolf DC Olson A Bao W Hanauer SB Comparison of scheduled and episodic treatment strategies of infliximab in Crohn’s disease Gastroenterology 2004 126 402 413 10.1053/j.gastro.2003.11.014 14762776

[B3] Hanauer SB Top-down versus step-up approaches to chronic inflammatory bowel disease: presumed innocent or presumed guilty Nat Clin Pract Gastroenterol Hepatol 2005 2 493 10.1038/ncpgasthep0318 16355136

[B4] Teague M Baddour LM Wruble LD Liver abscess: a harbinger of Crohn’s disease Am J Gastroenterol 1988 83 1412 1414 3195548

[B5] Heneghan HM Healy NA Martin ST Ryan RS Nolan N Traynor O Waldron R Modern management of pyogenic hepatic abscess: a case series and review of the literature BMC Res Notes 2011 4 80 10.1186/1756-0500-4-80 21435221 PMC3073909

[B6] Sharara AI Rockey DC Pyogenic liver abscess Curr Treat Options Gastroenterol 2002 5 437 442 10.1007/s11938-002-0031-0 12408780

[B7] Malik AA Bari SU Rouf KA Wani KA Pyogenic liver abscess: changing patterns in approach World J Gastrointest Surg 2010 2 395 401 21206721 10.4240/wjgs.v2.i12.395 PMC3014521

[B8] Kreuzpaintner G Schmidt WU West TB Tischendorf FW Two large liver abscesses complicating Crohn’s disease Z Gastroenterol 2000 38 837 840 10.1055/s-2000-9996 11089268

[B9] Baca B Hamzaoğlu I Karahasanoğlu T Hamzaoğlu HO Laparoscopic treatment of pyogenic liver abscess complicating Crohn’s disease: a case report Turk J Gastroenterol 2007 18 58 61 17450499

[B10] Patel TR Patel KN Boyarsky AH Staphylococcal liver abscess and acute cholecystitis in a patient with Crohn’s disease receiving infliximab J Gastrointest Surg 2006 10 105 110 10.1016/j.gassur.2005.04.006 16368499

[B11] Karaca C Güler N Yazar A Camlica H Demir K Yildirim G Liver abscess as a rare complication of Crohn’s disease: a case report Turk J Gastroenterol 2004 5 45 48 15264121

[B12] Zakout R Fonseca M Santos JM Marques A Távora I Oliveira E Ferreira C Victorino RM Multiple aseptic liver abscesses as the initial manifestation of Crohn’s disease: report of a case Dis Colon Rectum 2009 52 343 345 10.1007/DCR.0b013e318199db60 19279433

[B13] Greenstein AJ Sachar DB Lowenthal D Goldofsky E Aufses AH Jr Pyogenic liver abscess in Crohn’s disease Q J Med 1985 56 505 518 4048390

[B14] Papavramidis TS Sapalidis K Pappas D Karagianopoulou G Trikoupi A Souleimanis CH Papavramidis ST Gigantic hepatic amebic abscess presenting as acute abdomen: a case report J Med Case Rep 2008 2 325 10.1186/1752-1947-2-325 18847505 PMC2572068

[B15] Appau KA Fazio VW Shen B Church JM Lashner B Remzi F Brzezinski A Strong SA Hammel J Kiran RP Use of infliximab within 3 months of ileocolonic resection is associated with adverse postoperative outcomes in Crohn’s patients J Gastrointest Surg 2008 12 1738 1744 10.1007/s11605-008-0646-0 18709420

[B16] Kirsner JB Shorter RG Recent developments in nonspecific inflammatory bowel disease (second of two parts) N Engl J Med 1982 306 837 848 10.1056/NEJM198204083061404 7038490

[B17] Smith BM Zyromski NJ Allison DC Community-acquired methicillin-resistant *Staphylococcus aureus* liver abscess requiring resection Surgery 2007 141 110 111 10.1016/j.surg.2006.05.010 17188175

[B18] Hope WW Vrochides DV Newcomb WL Mayo-Smith WW Iannitti DA Optimal treatment of hepatic abscess Am Surg 2008 74 178 182 18306874

[B19] Cerwenka H Pyogenic liver abscess: differences in etiology and treatment in Southeast Asia and Central Europe World J Gastroenterol 2010 28 2458 2462 10.3748/wjg.v16.i20.2458 PMC2877174 20503444

[B20] Aydin C Piskin T Sumer F Barut B Kayaalp C Laparoscopic drainage of pyogenic liver abscess JSLS 2010 14 418 420 10.4293/108680810X12924466006567 21333200 PMC3041043

[B21] Hsieh HF Chen TW Yu CY Wang NC Chu HC Shih ML Yu JC Hsieh CB Aggressive hepatic resection for patients with pyogenic liver abscess and APACHE II score > or =15 Am J Surg 2008 196 346 350 10.1016/j.amjsurg.2007.09.051 18718219

[B22] Gould IM Cauda R Esposito S Gudiol F Mazzei T Garau J Management of serious methicillin-resistant *Staphylococcus aureus* infections: what are the limits? Int J Antimicrob Agents 2011 37 202 209 10.1016/j.ijantimicag.2010.10.030 21300528

